# My Work Is Meaningless: The Consequences of Perceived Occupational Stigma for Employees in High-Prestige Occupations

**DOI:** 10.3389/fpsyg.2022.715188

**Published:** 2022-04-27

**Authors:** Bo Huang, Lina Ma, Li Huang

**Affiliations:** ^1^School of Labor and Human Resource, Renmin University of China, Beijing, China; ^2^School of Education Science, Sichuan Normal University, Chengdu, China; ^3^Business School, Yunnan University of Finance and Economics, Kunming, China

**Keywords:** occupational stigma, meaningfulness, withdrawal, job embeddedness, high-prestige workers, preschool teachers

## Abstract

Occupational stigma is pervasive, but there is a lack of understanding about how it impacts the behaviors of employees in relatively high-prestige occupations. We draw on the job characteristics model and social information processing theory to establish hypotheses about the effects of occupational stigma on the withdrawal behavior of employees in a relatively high-prestige occupation (preschool teacher). We suggest that perceptions of skill variety and task significance among high-prestige employees may be negatively influenced due to occupational stigma perception. In addition, occupational stigma conveys information to employees that the work they do is not appreciated by beneficiaries. For those reasons, making it difficult for them to perceive the meaningfulness of their work. This lack of meaningful experience is in turn positively associated with employees’ withdrawal behavior. Furthermore, we propose that these indirect effects are moderated by perceived job embeddedness of employees. Based on data collected at two time points from 466 preschool teachers in China, we find that occupational stigma is positively related to employees’ withdrawal behavior through meaningfulness. In addition, the negative relationship between perceived occupational stigma and experienced meaningfulness is stronger for employees with high job embeddedness than for employees with low job embeddedness.

## Introduction

“A New York-listed Chinese education company was under police investigation on Friday after accusations of suspected child abuse at one of its Beijing kindergartens set off a social media firestorm.” (Mitchel and Liu, 2017).

Jobs such as teacher, doctor, and scientist have long been considered the most prestigious professions in China (Lin and Xie, 1988; Chen et al., 2021). In recent years, however, there has been significant negative press about such professions. When negative press builds over time, the profession is likely to become stigmatized in the eyes of the public and viewed as sinful or deceptive (Hughes, 1951; Ashforth and Kreiner, 1999, 2014). Occupational stigmatization has become an increasing problem, with a constant rise in violence against workers whose occupations have become stigmatized (Wang et al., 2021) and a consequent negative impact on perceptions of the industry as a whole industry (Brien et al., 2017). When employees are aware that their work has become stigmatized and believe others view them negatively because of the work they do, this is referred to as occupational stigma consciousness (Pinel, 1999; Pinel and Paulin, 2005; Shantz and Booth, 2014).

Studies have found that employees who perceive themselves to face occupational stigma experience several negative outcomes (see Kreiner and Mihelcic, 2020; Zhang et al., 2021; Kreiner et al., 2022, for review), such as higher occupational and organizational disidentification (Lai et al., 2012; Schaubroeck et al., 2018), lower job satisfaction (Baran et al., 2012), increased organizational production deviance behaviors (Shantz and Booth, 2014), and higher turnover intention (Pinel and Paulin, 2005; Lopina et al., 2012; Schaubroeck et al., 2018). However, other researchers have suggested that perceiving occupational stigma may facilitate more advantageous judgments of the employees themselves and their work, which will lead to positive outcomes (Helms and Patterson, 2014).

Despite the considerable attention scholars have devoted to examining occupational stigma, our theoretical understanding of occupational stigma is far from complete. Ashforth and Kreiner (1999) highlighted a need to understand occupational stigma with reference to occupational prestige (relatively low vs. relatively high) because occupational prestige represents social perceptions of different kinds of work and likely influences the social construction of occupations. However, existing research has primarily focused on typically low-prestige occupations that are stigmatized by the majority of society (Ashforth et al., 2017; Schaubroeck et al., 2018; Zhang et al., 2021), such as casino employees (Lai et al., 2012), exotic dancers (Grandy, 2008), animal shelter workers (Baran et al., 2012), and garbage collectors (Peter et al., 2019). Occupational stigma may also be directed at high-prestige occupations, such as physicians or teachers. Thus, there is a need to further explore the influence of occupational stigma on employees whose occupational prestige is relative higher than those in low-prestigious occupations (Baran et al., 2012; Ashforth and Kreiner, 2014). For example, Ashforth and Kreiner (2014) have strongly call for sampling from a variety of occupations (p. 100). This is important because scholars have suggested that all occupations occasionally involve some form of occupational stigma, and a holistic understanding of the influencing processes related to various kinds of occupations is needed (Hughes, 1951; Kreiner et al., 2006). Moreover, studies regarding occupational stigma have been largely qualitative, and scholars have called for further research to explore occupational stigma via quantitative methods (Ashforth et al., 2017; Schaubroeck et al., 2018; Zhang et al., 2021). Finally, Ashforth and Kreiner (1999) suggested that prestige could serve as “status shield” to buffer some of the negative influence of occupation stigma. However, whether the shield effect actually occurs remains lack of understanding from an empirical perspective.

Given the inconsistent and inadequate findings on occupational stigma, the job characteristics model (JCM, Hackman and Oldham, 1976), as well as social information processing (SIP, Salancik and Pfeffer, 1978), have been adopted to explain how, why, and when occupational stigma has a negative impact on the withdrawal of high-prestige occupation employees. Withdrawal is a mixture of behaviors that reflect attempts to psychologically disengage from tasks, such as lateness and deliberately lowering work effort (Hanisch and Hulin, 1990; Spector et al., 2006). Withdrawal can have highly negative effects on organizations and occupations; thus, we consider this in more detail in the next section.

**FIGURE 1:**
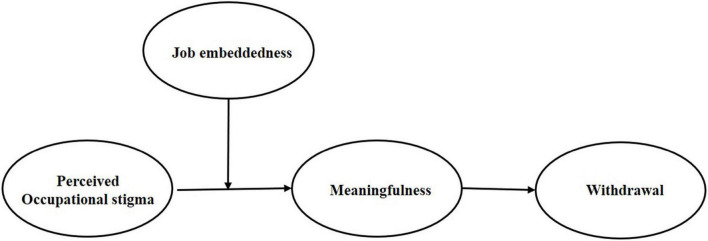
Theoretical model of the current research.

Briefly stated, JCM suggests that certain objective job characteristics can impact employees’ psychological state (e.g., experienced meaningfulness of the work) and influence work results (Hackman and Oldham, 1976). SIP indicates that job characteristics are subjective judgments that are socially constructed from social cues (Salancik and Pfeffer, 1978). We integrate JCM and SIP to propose that occupational stigma undermines employees’ perceptions about the skill variety and task significance within their job. In addition, occupational stigma also signals the fact that employees’ work is not appreciated by the beneficiaries. Such a lack of skill variety and significance or value perception about the job makes it difficult for employees to perceive meaningfulness in their work, leading them to avoid the unfavorable workplace as it cannot meet their need to achieve meaningful action. As a result, employees’ well-being and productivity are reduced (Robinson and Bennett, 1997). Moreover, our framework provides a more complete understanding of occupational stigma by examining the moderating role of job embeddedness. SIP suggests that if a plausible explanation for behaviors is available, favorable attitudes are less likely to develop (Salancik and Pfeffer, 1978, p. 237). Consistent with this idea, we theorize that the effect of occupational stigma on employees’ experience of meaningfulness will be moderated by job embeddedness, and ultimately be associated with employees’ withdrawal behavior. The more employees are embedded in their current job, the more the meaningfulness they derive from their work will suffer when they perceive occupational stigma, which will be positively associated with withdrawal behavior. Figure 1 illustrates our theoretical model.

The present study makes several contributions to the literature. First, given that the majority of research on occupational stigma has exclusively examined low-prestige occupations workers using qualitative research methods, we advance the topic by examining the influence of occupational stigma on relatively high-prestige occupations using a quantitative approach. In addition, by challenging the dominant assumption that job embeddedness is entirely beneficial, our research adds evidence on the potential downside effect of embeddedness on employees (Ng and Feldman, 2012; Allen et al., 2016). Further, by examining the negative impact of occupational stigma and the moderating role of job embeddedness on the experience of meaningfulness, we respond to recent calls to uncover the factors that impede or challenge the process by which individuals experience work as meaningful (Lysova et al., 2019). Our study also contributes to the literature by integrating the JCM (Hackman and Oldham, 1976) and SIP (Salancik and Pfeffer, 1978) theories to provide a framework for understanding occupational stigma, which has mainly been discussed with reference to social identity theory to date (e.g., Baran et al., 2012; Schaubroeck et al., 2018).

## Literature Review

### The Job Characteristics Model and Social Information Processing Theory

The JCM was developed by Hackman and Oldham (1976). It suggests that when a job has certain characteristics, such as task significance, it can improve employees’ psychological state (e.g., experienced meaningfulness of the work) and thus improve their work results (performance, job satisfaction, etc.). As JCM suggests, these job characteristics are objective; and as long as tasks contain these characteristics, they will produce corresponding effects on employees. However, employees do not work in a vacuum but rather in a social context; SIP theory brings this aspect under consideration. Empirical research has also indicated that task significance is not entirely generated from objective job characteristics; social information and cues also play an important role (Wrzesniewski et al., 2003; Grant, 2008).

Social information processing (Salancik and Pfeffer, 1977, 1978) has been widely used for interpreting employees’ work attitude and behaviors (e.g., Yam et al., 2018; Yang et al., 2019). SIP theory suggests that employees’ perceptions, attitudes, and behaviors are not given, but rather are products of information processing activities (Zalesny and Ford, 1990). There are two foundational ideas of SIP. First, it is possible for employees to make use of social information or cues to derive meaningfulness, a sense of importance, and variety from the jobs. Second, information comes from a wide range of sources, including the worker’s own past experience, others’ feelings about the job, the behavioral responses of others, and information about features of the environmental context. Salancik and Pfeffer (1978, p. 230) suggested that “the information used can be any information.”

Based on the core ideas of JCM and SIP, we believe that these perspectives are particularly suited to exploring the effect that occupational stigma has on employees. Primarily, both JCM and SIP share the assumption that when perceptions of job characteristic are higher workers are expected to be more productive, and vice versa (Hackman and Oldham, 1976; Salancik and Pfeffer, 1978). More specifically, both theoretical perspectives suggest that when employees perceive their occupations as having higher key job characteristics (e.g., task significance) they will experience their work as more meaningful and thus improve their work outcomes (Grant, 2008). Thus, JCM and SIP provide an overarching theoretical basis for our study. However, the focus of JCM vs. SIP differs, since they each emphasize different antecedents of job characteristics. Specifically, JCM emphasizes the objective, and SIP the subjective or perceived, job characteristics (Hackman and Oldham, 1976; Salancik and Pfeffer, 1978; Blau and Katerberg, 1982). Researchers have suggested that employees use both objective and subjective information to construct their job characteristics in reality (Griffin, 1983; Thomas and Griffin, 1983). Thus, JCM and SIP are both compelling and complement each other in explaining the source of task characteristics, hence their frequent integration as theoretical grounding for research (e.g., Grant, 2008).

### Occupational Stigma, Meaningfulness, and Withdrawal Behavior

#### Occupational Stigma

Goffman (1963) pioneering analysis of stigma is often regarded as the starting point for stigma research. Goffman (1963) defined stigma as a certain person or group being humiliated and belittled by society, and divided stigma into three categories: bodily, character-based, and tribal (for a review see Summers et al., 2018). In the 1950s, the term “dirty work” was used to refer to occupations that are generally considered humiliating, demeaning, or sickening to the individuals and groups engaged in them, and dirty work was classified as physically, socially, and morally tainted (Hughes, 1951, 1958).

Connected to these pioneering works, organizational behavior scholars began to explore the occupational stigma problem (e.g., Ashforth and Kreiner, 1999; Kreiner et al., 2006; Ashforth et al., 2007). Combining Hughes (1958) classification of “taint” with two levels of occupational prestige (relatively low and relatively high), Ashforth and Kreiner (1999) divided occupational stigma into six categories, and offered criteria for the three forms of taint: physical taint refers to occupations connected to uncomfortable objects or concepts, such as junk or death (e.g., garbage collectors, funeral directors) or work that takes place under especially poisonous or unsafe conditions (e.g., firefighting); social taint refers to occupations that involve frequent connection to stigmatized individuals or groups (e.g., prison guards) or involves a servile relationship to others (e.g., massage therapists); and moral taint is connected with occupations viewed as immoral or of dubious virtue by the majority of people in society (e.g., exotic dancers), or as frequently utilizing dishonest or intrusive methods (e.g., telemarketers).

Although Kreiner et al. (2006) have suggested that all occupations occasionally involve some form of occupational stigma, and Hughes (1951) also emphasized that “dirty work of some kind is found in all occupations,” we feel it is necessary to explain in detail why we suggest that the role of preschool teacher is at risk of facing occupational stigma. First, preschool teachers mainly work with children aged 3–6, so their work environment is usually noisy (e.g., children’s screaming and crying) and typically deals with bodily excretions (i.e., involves uncomfortable objects) (Gu et al., 2020). Hence, we posit that preschool teacher can be seen as a physically tainted occupation. Moreover, in addition to teaching tasks such as training children in language and helping children to develop their social and emotional skills, preschool teachers are required to engage in non-teaching tasks and child care responsibilities, such as dressing and arranging lunch (i.e., involves servile relationship to others, Gu et al., 2020). Accordingly, preschool teacher can be viewed as a socially tainted occupation. More importantly, as illustrated above, preschool teachers have been subject to negative press in recent years for child abuse behaviors, not only in China but also in other countries (e.g., Whitted and Dupper, 2008). Child abuse is seen as highly sinful and unforgivable for the negative effects it imposes on children’s mental health and development (Stoltenborgh et al., 2011). As a result, preschool teachers may also be at risk of being morally stigmatized. Indeed, while child abuse is an isolated phenomenon, and the vast majority of preschool teachers would never commit it or even witness it without reporting it, the public may misperceive the occupation due to the frequent reports they read about misconduct related to the role (Vough et al., 2013). Other examples of roles that have been stigmatized by misconduct by the minority include members of the clergy, which have recently been stigmatized by Catholic Church sex scandals (Piazza and Jourdan, 2018), and auditors, who have been stigmatized by the Enron scandal (Jensen, 2006).

#### The Negative Effect of Occupational Stigma on Meaningfulness

Meaningfulness is the degree to which employees find their job meaningful, significant, and worthwhile (Hackman and Oldham, 1976). Based on JCM and SIP theory, we argue that when employees of high-prestige occupations perceive occupational stigma their perceived meaningfulness from their job will be undermined. Since JCM focuses on objective and SIP on subjective elements regarding how individuals perceive their environment, our augment unfolds along two lines.

With respect to the objective side, JCM suggests that job characteristics produce psychological states among employees and ultimately lead to a series of job outcomes (Hackman and Oldham, 1976). More specifically, three core job dimensions—skill variety, task identity, and task significance—combined cumulatively to impact the meaningfulness of a job (Hackman and Oldham, 1976; Morgeson and Campion, 2003). When a job involves the use of employees’ extensive variety of skills and abilities, employees are likely to have perceptions of skill variety. Task significance is the extent to which one’s task has a considerable positive influence on the lives or work of others (Hackman and Oldham, 1976). If employees perceive themselves to be subject to occupational stigma, it is likely that their job can be said to involve some level of dirtiness; this may decrease the employees’ perception of key job characteristics, as per JCM, consequently obstructing the experience of meaningfulness.

Taking preschool teacher as an example, as argued above this occupation likely faces the risk of being physically and socially stigmatized due to aspects such as noise and the need to complete low-skilled tasks (dressing, toileting, facilitating lunch, etc.). These low-skilled tasks may undermine preschool teachers’ feelings regarding skill variety, and may make them feel akin to babysitters since they may think that these low-skilled, non-teaching tasks are irrelevant to the children’s development, growth, and future social and academic success. In addition, these low-skill tasks do not fully utilize teachers’ skills and abilities. Indeed, studies have found that employees who feel they are overqualified for their job experience cynicism and doubt the meaningfulness of their job (Luksyte et al., 2011). In this vein, preschool teachers possess a wealth of teaching skills but are inevitably required to perform low-skilled tasks. Therefore, we posit that high-prestige occupation employees who perceive a higher level of occupational stigma may be less likely to experience the key job dimensions of skill variety and task significance, which will ultimately negatively influence their perceived meaningfulness of their job. The reasoning above is based on the objective view—that is, perspectives that preschool teachers may derive directly from the job itself. However, as suggested in previous research, dirtiness is not inherent in the work itself but is a social construct that is imposed by others (Ashforth and Kreiner, 1999). Thus, in addition to those objective job characteristics preschool teachers may experience, it is also necessary to focus on the subjective—that is, social—influence they may encounter.

With regard to the subjective viewpoint, SIP suggests that the characteristics of work are not inherent but are constructed from social information, and that perceptions of job features may be influenced by factors other than the objective characteristics of the work (Salancik and Pfeffer, 1978). Accordingly, we argue that the perception of the key job dimension task significance may be undermined by the influence of occupational stigma. Specifically, we posit that occupational stigma may convey information or cues to employees of high-prestige occupations that their work is not appreciated by the beneficiaries of their work; as a result, it is difficult for jobholders to perceive the significance of their work. The meaning of work is usually enhanced when individuals feel that their work effort significantly and positively impacts others (Hackman and Oldham, 1976). Through three time points over a 6th-month period longitudinal research, Blake (2017) found that task significance consistently predicted meaningful work.

Grant (2008) outlined two main effects of task significance on employees. The first is *perceived social impact*, which describes the extent to which employees maintain that their own working activities could improve the welfare of others (Grant, 2007)—for example, when teachers or doctors (representing relatively high-prestige occupations) perceive that their work has a social impact, or know their work affects the development of students or the lives of patients, respectively. The second mechanism is *perceived social worth*, which describes the degree to which employees feel other people appreciate their work (Baumeister and Leary, 1995; Ashforth and Kreiner, 1999; Leary and Baumeister, 2000; Elliott et al., 2005; Grant, 2008). The effect of task significance on jobholders is influenced by perceptions of both social impact and social worth. However, Grant (2008) suggested that there is difference between perceived social impact and perceived social worth. Having a positive impact on beneficiaries does not necessarily equate to appreciation for employees’ efforts, although jobholders may perceive social impact and believe that their work has a positive influence on others’ welfare, they may not always perceive social worth. In other words, they may not believe other people appreciate their work. Indeed, recipients may not only be ungrateful for their work but may be hostile or aggressive (e.g., Zapf, 2002), and can even seek to hurt them, especially under the influence of occupational stigma (e.g., Wang et al., 2021). Therefore, when employees of high-prestige occupations perceive their job to be subject to occupational stigma, they may perceive information or cues that the recipients of their work do not appreciate their efforts, which makes it more difficult for them to perceive task significance, and in turn reduces their perceived meaningfulness. For example, Grandey et al. (2004) found that employees who report greater aggression from the beneficiaries of their work experience more emotional exhaustion and are more likely to be absent from work.

To sum up, from the perspectives of JCM and SIP, the job dimensions skill variety and task significance are crucial for employees to perceive meaningfulness, since perceptions of skill variety and task significance among high-prestige employees may be negatively influenced due to occupational stigma perception. In addition, occupational stigma may deliver cues or information to employees about a lack of social worth (i.e., that their work is not appreciated by the beneficiaries thereof). Consequently, meaningfulness may be undermined. Thus, based on the reasoning and literature described above, we hypothesize the following:

***H1.* Perceived occupational stigma has a negative relationship with experienced meaningfulness.**

#### The Positive Effect of Occupational Stigma on Withdrawal Through Meaningfulness

Through its effects on employees’ experience of meaningfulness, occupational stigma perception is likely to increase employees’ withdrawal behavior. Withdrawal has been defined as avoiding one’s job tasks while at the same time maintaining organizational membership (Hanisch and Hulin, 1990). Withdrawal entails poor performance and deviant organizational functioning (Robinson and Bennett, 1995); thus, withdrawal is often considered a critical indicator in organizational research (Harrison et al., 2006; Kammeyer-Mueller et al., 2013). Moreover, Kammeyer-Mueller and Wanberg (2003) proposed that withdrawal is more harmful for the organization compared to other deviant behaviors. For example, physicians must carefully prepare for operations, while preschool teachers are often required to guide groups of children on outdoor teaching activities; these tasks require a highly degree of concentration and energy, meaning that if employees enact withdrawal behaviors or invest insufficient effort it may lead to errors or accidents. These negative outcomes may further worsen stigmatization of the occupation.

Researchers have suggested that employees who experience disagreeable characteristics or negative events at work may withdraw from these unfavorable work conditions (Kathy et al., 1998). For example, when work is perceived boring by employees it will lead to withdrawal (Debus et al., 2020). Based on a study of call center employees, Shantz and Booth (2014) found that perceived occupational stigma is significantly correlated with organizational production deviant behaviors for employees with high core self-evaluation; however, the study did not explore the mechanism underlying the process. Further, Schaubroeck et al. (2018) found that when employees perceive higher levels of dirtiness than usual it results in a higher level of occupational disidentification and in turn leads employees to exhibit withdrawal behaviors. This study provided further insight into the consequences and mechanism of occupational stigma, but the employees were sampled from a broad range of occupations rather than being sampled on the basis of public stigma. Consistent with prior research (e.g., Shantz and Booth, 2014; Schaubroeck et al., 2018) and due to the especially negative outcomes withdrawal behavior may bring to the organization and occupation, we decided to further explore the mechanism underlying occupational stigma and withdrawal, as well as the boundary conditions of this process.

According to JCM, when job dimensions (e.g., significance) that lead to employees experiencing meaningfulness are high (vs. low), employees’ work motivation will increase (vs. decrease), and several positive (vs. negative) outcomes (e.g., performance, well-being) can be expected (Hackman and Oldham, 1976). SIP theory also suggests that individuals’ perceptions, attitude, and behavior can be influenced by social information. Attitude, which is constructed from social information, mediates the relationship between social information and behavior (Salancik and Pfeffer, 1978; Zalesny and Ford, 1990). If employees perceive that their work is not meaningful as the result of occupational stigma, they are less likely to work with vigor. The key point here pertains to the socially constructed experience or perception that the work is unvalued, unimportant, or worthless. Researchers have suggested that people are motivated to understand whether their actions are meaningful (Shamir, 1991; Wrzesniewski et al., 2003). Surveys have also shown that, relative to promotions, wage, and job security, meaningful work is the most valued job feature (Cascio, 2003). Individuals are primarily motivated by the pursuit of social value (Baumeister and Leary, 1995; Ryan and Deci, 2000). If individuals perceive their endeavor as worthwhile or highly regarded, they are more likely to work hard (Rosen et al., 1987; Rhoades and Eisenberger, 2002). Empirical studies have extensively investigated the positive influence of meaningfulness perception on employees’ motivation (e.g., Hackman and Oldham, 1976; Lips-Wiersma and Wright, 2012), work engagement (e.g., Ahmed et al., 2016), in-role and out-role performance (e.g., Grant, 2007, 2008; Lam et al., 2016) and job satisfaction (e.g., Fairlie, 2011). In contrast, employees that experience less meaningfulness in their work may feel bored and worthless, which is detrimental to personal well-being (e.g., Van Selm and Dittmann-Kohli, 1998; Lam et al., 2016). Even under the “shelter” of prestige, employees are likely to experience burnout when they feel meaningless (e.g., Shanafelt et al., 2009). Therefore, the lack of motivation due to the meaninglessness of the work, as well as the inclination to avoid an unfavorable workplace that cannot meet the need for meaning, will lead employees to be more likely to engage in withdrawal behaviors. Thus, we hypothesize the following:

***H2.* Experienced meaningfulness mediates the relationship between perceived occupational stigma and withdrawal behavior**

### Moderating Role of Job Embeddedness

In this section, we develop the hypothesis that job embeddedness strengthens the negative association between occupational stigma perception and experience of job meaningfulness and withdrawal. The central logic underlying this hypothesis is that behavior can serve as a source of information for constructing attitude (Salancik and Pfeffer, 1978; Zalesny and Ford, 1990). People justify their behavior in order to make that behavior meaningful and explainable (Salancik and Pfeffer, 1978). However, a reasonable justification for behavior may reduce the intrinsic value of a task, producing a more negative attitude than if no such justification were provided (Salancik and Pfeffer, 1978). We provide more detail on this below.

Job embeddedness, which is defined as “the combined forces that keep a person from leaving his or her job” (Yao et al., 2004, p.159), comprises three dimensions: links, fit, and sacrifice (Mitchell et al., 2001). When an employee is embedded in their organization, it means they have a global sense that it would be difficult to quit. Employees may feel connected to their working team, fit with the culture or work requirements, or perceive a cost to be incurred with regard to some benefits (e.g., salary, advancement opportunities, or stability) if they leave the organization. That is, job embeddedness may provide employees with a reasonable explanation for their retention and work effort. For example, a preschool teacher may stay at a kindergarten due to the high salary offered, or the idea that their skills are not suited to other occupations, and so on.

As described by SIP theory, behavior is an undeniable aspect of each individual’s world, and is based on their behavior, individual constructs, or interpretations of features of the environment, which lead to attitude formation. Like a player in a symphony orchestra, the music the individual plays become part of their environment (the background), influencing their subsequent attitudes and actions. Similarly, if an employee is embedded in an organization, their retention, or their effort devoted to the work, becomes an inseparable part of their world and in turn impacts their attitudes (e.g., meaningfulness). In addition, people seek to rationalize their actions in order to make them meaningful and explainable (Salancik and Pfeffer, 1978). Thus, employees in a stigmatized occupation may feel the need to justify their reasons for staying. However, no matter what their justification is in this regard, at any one point in time only one reason is salient. As Salancik and Pfeffer (1978, p.237) explained, “If a person is thinking of one thing, he or she cannot simultaneously think of something else. Thus, salient information not only provides an explanation for behavior but also interferes with the use of other information to construct other explanations.” According to this reasoning, an employee must be able to answer the question, “Why don’t I quit such a stigmatized occupation?” The answer for an embedded employee may be obvious—the cost if they leave (e.g., salary). This explanation is reasonable enough for embedded employees; however, SIP suggests that a reasonable justification for behavior may reduce the intrinsic value of a task, producing a more negative attitude than if no such justification were provided (Salancik and Pfeffer, 1978). For example, Pallak et al. (1974) found that subjects who were told that their work was worthless reported a boring task to be more enjoyable than did subjects who were not told this, or who had to accomplish this boring task as a course requirement. Therefore, although an embedded employee has sufficient reasons to explain their reasons for remaining in a stigmatized occupation, their perceptions of meaningfulness may be undermined. However, for unembedded employees who have no reasonable external explanation for their retention, this may be attributed to internal factors (e.g., the job is meaningful), such that perceived meaningfulness may be less likely to be influenced. As SIP suggested that if employees engage in some behavior without socially recognized rewards, sanctions, or other external pressures, they will rationalize it with reference to personal motivations, attitudes, and needs (Salancik and Pfeffer, 1978).

In brief, consistent with SIP theory and empirical findings, we therefore suggest that the meaningfulness perceived by employees is more likely to be undermined when the employee is embedded in the job compare to when they are not. Embedded employees may already have plausible justification for their activities when they perceive occupational stigma, since it is reasonable to attribute their staying in the occupation to their connection to, fit with, or sacrifice due to their job (Lee et al., 2014; Kiazad et al., 2015). In contrast, meaningfulness perceived by unembedded employees is less likely to be undermined due to the lack of socially justified reasons to stay, and they may tend to explain their staying in the occupation with reference to the positive characteristics (e.g., meaningfulness) of their job. These arguments are consistent with studies which have shown that employees exhibit more negative attitudes when situations are sufficiently justified (Zanna, 1973; Salancik, 1974). Thus, we hypothesize:

***H3:* Job embeddedness moderates the relationship between perceived occupational stigma and experienced meaningfulness, such that the negative relationship is stronger when employees are more embedded in their job.**

According to the analytical framework of moderated mediation, job embeddedness has a significant moderating effect on the path of perceived occupational stigma to experienced meaningfulness (H3), and perceived occupational stigma has a mediating effect on withdrawal through experienced meaningfulness (H2); in such situations, a moderated mediation model should typically be created. Specifically, based on JCM and SIP theory, when employees are highly embedded in their organization their experienced meaningfulness is more likely to be undermined, which leads to higher levels of withdrawal behavior. Consequently, experienced meaningfulness plays a more crucial role in bridging the effects of perceived occupational stigma on withdrawal behaviors. Conversely, when job embeddedness is low, employees’ experienced meaningfulness is less likely to be undermined, which leads to low level of withdrawal behaviors. Experienced meaningfulness accordingly plays a less significant role in transmitting the influence of perceived occupational stigma on withdrawal behaviors. Taken together, we offer our final hypothesis:

***H4:* Job embeddedness moderates the indirect relationship between perceived occupational stigma and withdrawal, such that the indirect relationship is stronger when employees are more embedded in their job.**

## Materials and Methods

### Sample and Data Collection

Our hypotheses were tested using a two-wave field study of full-time preschool teachers in China. This was an excellent occupation for a test of our hypotheses for two reasons. First, teaching is traditionally viewed as a highly prestigious occupation in China (Lin and Xie, 1988; Chen et al., 2021). Through teaching tasks such as training children in language and helping children to develop their social and emotional skills, empirical studies have demonstrated that preschool teachers’ work is key for children’s development, growth, and future social and academic success (Siekkinen et al., 2013; Gu et al., 2020). Specifically, based on occupational prestige scores (Smith and Son, 2012), preschool teacher has higher prestige scores (5.3) compared to the other occupations (e.g., Table Clearer, 2.3; Garbage Collector, 3.8) which typically been studied in occupational stigma literature (Peter et al., 2019). Second, depending on the conceptualization of dirty work (Ashforth and Kreiner, 1999), the occupation of preschool teacher may entail a risk of being physically, socially, and morally stigmatized as mentioned above.

We contacted 1,500 full-time preschool teachers from different cities in northern, central, and southwestern China to participate in this research. When we first contacted the participants, we provided an overview of our study (e.g., occupation-related research) but the participants were not given any specific research hypotheses. A total of 466 valid questionnaires were collected, yielding a response rate of 31.1%. Respondents comprised 463 women (99.4%). The average length of teaching was 7.28 years (*SD* = 8.82), the average age was 30.21 years (*SD* = 8.18), and 304 (65.24%) held a bachelor’s degree or higher.

A time-lagged research design was used; that is, data were collected in two stages to reduce common method bias (Podsakoff et al., 2003). We offered incentives for participation to maximize the response rate. The teachers were asked to report their perception of occupational stigma, job embeddedness, and demographic information at Time 1. 1 month later, at Time 2, the teachers were asked to completed measures of perceived meaningfulness of their job and self-reported withdrawal behavior. Each participant was compensated with 15 RMB (approximately 2 USD) per survey.

### Measurement Instruments

All items detailed below underwent the standard back-translation process recommended by Brislin (1980) to ensure that all survey materials were accurately translated from English to Chinese. See Appendix Table 1 for sample measurement items.

We used the six-item scale developed by Pinel (1999) to measure perceived occupational stigma. Consistent with previous studies (Pinel and Paulin, 2005; Shantz and Booth, 2014), the instructions and items pertain to how people who are not preschool teachers think about and interact with preschool teachers. Items were scored on a 5-point Likert scale ranging from 1 (strongly disagree) to 5 (strongly agree). The Cronbach’s alpha for this scale was 0.81.

We adapted Crossley et al.’s (2007) 7-item scale to measure job embeddedness, as this has been found to be appropriate for us in the Chinese context (e.g., Lyu and Zhu, 2019). Answers were given on a 7-point Likert scale ranging from 1 (strongly disagree) to 7 (strongly agree). The Cronbach’s alpha for this scale was 0.89.

We used the 10-item Work as Meaning Inventory (WAMI) originally developed by Steger et al. (2012) to measure meaningfulness. This scale has demonstrated good validity in the context of China (e.g., Zhang et al., 2020). The WAMI consists of three dimensions: positive meaning, meaning-making, and greater-good motivations. Items were scored on a 5-point Likert scale ranging from 1 (absolutely untrue) to 5 (absolutely true). The Cronbach’s alpha for this scale was 0.89.

We used the On-the-Job Behavior Scale developed by Lehman and Simpson (1992) to measure teachers’ withdrawal behavior. This scale has again demonstrated good validity with respect to a Chinese sample (e.g., Yuan et al., 2021). The scale includes four dimensions: positive work behavior, psychological withdrawal behavior, physical withdrawal behavior, and oppositional work behavior. In the current study, psychological withdrawal (eight items) and physical withdrawal (four items) dimensions were included. Items were scored on a 7-point Likert scale ranging from 1 (never) to 7 (very often) based on how often the behaviors had occurred in the previous 12 months. The Cronbach’s alpha for this scale was 0.89.

Several control variables were included in this study. We controlled for employees’ age and tenure (in years) to exclude possible confounding factors when predicting withdrawal behaviors, because it has been shown that higher age and work experience correlate with lower levels of withdrawal or turnover intention (Knudsen et al., 2007) as well as deviant behavior at work (Gruys and Sackett, 2010). Second, we controlled for employees’ income (in six categories) because income may impact job attitudes and withdrawal intentions. We also controlled for education (in four categories), because highly educated employees have been found to be less likely to exhibit deviant behavior at work (Gruys and Sackett, 2010). Finally, we did not control for gender because the vast majority (99.4%) of respondents were women.

## Results

### Preliminary Analyses

Before testing our hypotheses, we performed a series of confirmatory factor analyses to confirm the distinctive factor structure of our four variables. The hypothesized four-factor model—occupational stigma consciousness, job embeddedness, meaningfulness, and withdrawal—demonstrated a fairly good fit to the data (χ^2^/*df* = 2.95, CFI = 0.87, TLI = 0.86, RMSEA = 0.06, SRMR = 0.06; CFI and TLI were slightly below 0.90), and was also superior to three alternative models, including a three-factor model with occupational stigma consciousness and job embeddedness combined (χ^2^/*df* = 4.32, CFI = 0.78, TLI = 0.76, RMSEA = 0.09, SRMR = 0.09); a two-factor model with occupational stigma consciousness, job embeddedness, and meaningfulness combined (χ^2^/*df* = 7.06, CFI = 0.59, TLI = 0.56, RMSEA = 0.11, SRMR = 0.12); as well as a one-factor model (χ^2^/*df* = 9.24, CFI = 0.44, TLI = 0.40, RMSEA = 0.13, SRMR = 0.13). Therefore, the results provide support for the discriminant validity of measures. Descriptive statistics are presented in Table 1.

**TABLE 1:** Means, standard deviations, and correlations among variables.

Variables	*M*	*SD*	1	2	3	4	5	6	7	8
(1) Age	30.21	8.18								
(2) Education	2.59	0.66	−0.12*							
(3) Income	3.58	1.67	0.29**	0.47**						
(4) Tenure	7.28	8.82	0.84**	0.01	0.37**					
(5) POS	3.63	0.72	−0.11*	0.16**	0.02	–0.04	(0.81)			
(6) Job embeddedness	4.63	1.22	0.30**	−0.10*	0.18**	0.21**	−0.23**	(0.89)		
(7) Meaningfulness	3.85	0.55	0.12**	0.04	0.10*	0.08	−0.18**	0.30**	(0.89)	
(8) Withdrawal	2.09	0.83	−0.19**	0.25**	0.03	−0.09*	0.27**	−0.36**	−0.41**	(0.89)

n = 466; **p < 0.01, *p < 0.05; Cronbach’s alpha is in parentheses; POS = perceived occupational stigma.

### Hypothesis Testing

To test the hypotheses 1, linear regression was conducted. Perceived occupational stigma at Time 1 was negatively associated with experienced meaningfulness at Time 2 (β = –0.13, *p* < 0.001). To examine Hypotheses 2, a bootstrapping-based mediation test was conducted using an SPSS PROCESS macro with 2,000 resamples to produce a 95% confidence interval (CI; Hayes, 2013). Results revealed that perceived occupational stigma was associated with increased withdrawal behavior, mediated by experienced meaningfulness (β = 0.07, *SE* = 0.03, 95% CI = [0.03, 0.14], excluding 0). Hence, results provide support for Hypotheses 1 and 2.

**FIGURE 2:**
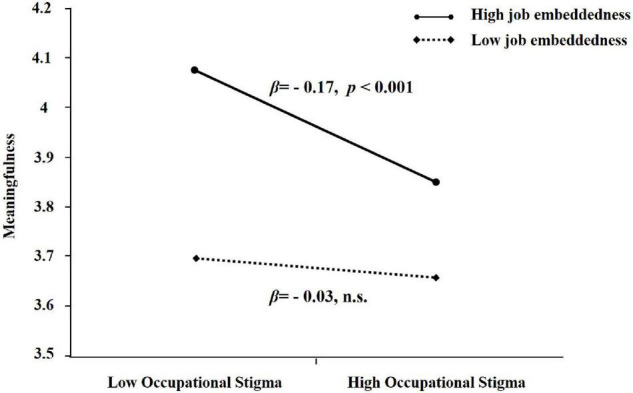
The interactive effect of perceived occupational stigma and job embeddedness on meaningfulness.

To test Hypothesis 3, the interactive effect of perceived occupational stigma and job embeddedness on experienced meaningfulness was examined. Results show that both perceived occupational stigma (β = –0.13, *p* < 0.001) and job embeddedness (β = 0.12, *p* < 0.001) were associated with experienced meaningfulness (Table 2, model 2 and 3). Further, results show that the interaction term (model 4) was significant (β = –0.06, *p* < 0.05) and the model explained significantly more variance after including the interaction term (R^2^ = 0.12, △R^2^ = 0.01, *p* < 0.05). To aid interpretation, we followed the recommendation by Aiken and West (1991) and plotted the interaction at values 1 SD above and below the mean of embeddedness, as shown in Figure 2. This effect was in the expected direction, such that the negative relationship between perceived occupational stigma and experienced meaningfulness was stronger when the level of job embeddedness was high (β = –0.17, *p* < 0.001) rather than low (β = –0.03, *n.s.*). Therefore, Hypotheses 3 is supported.

**TABLE 2:** Results of multiple regression analyses.

	Meaningfulness				Withdrawal		
	M1	M2	M3	M4	M5	M6	M7
Age	0.01*	0.01*	0.01	0.00	−0.03***	−0.02***	−0.02*
Education	0.03	0.06	0.08	0.08	0.31***	0.27***	0.30***
Income	0.02	0.02	0.00	0.00	–0.03	–0.03	–0.02
Tenure	–0.01	–0.01	0.00	0.00	0.02*	0.01	0.01
POS		−0.13***	−0.09**	0.16		0.25***	0.17***
Embeddedness			0.12***	0.32***			
POS × Embeddedness				−0.06*			
Meaningfulness							−0.56***
R^2^	0.02	0.05	0.11	0.12	0.10	0.14	0.27
△R^2^		0.03	0.06	0.01		0.04	0.13
F	2.79*	5.07***	9.78***	9.23***	12.56***	15.27***	28.96***
△F		13.89***	31.64***	5.33*		23.64***	83.65***

***p < 0.001, **p < 0.01, *p < 0.05; POS = perceived occupational stigma.

The PROCESS results show that the indirect effect of perceived occupational stigma on withdrawal via experienced meaningfulness was significant. The effect was significant when the level of job embeddedness was high (β = 0.10, *SE* = 0.03, 95% CI = [0.04, 0.16], excluding 0), but not when the level of job embeddedness was low (β = 0.02, *SE* = 0.04, 95% CI = [–0.05, 0.09], including 0). These findings support Hypothesis 4. In other words, although perceived occupational stigma is generally associated with increased withdrawal behavior, this effect is stronger when job embeddedness is high but disappears when job embeddedness is low. Together, our results suggest that perceived occupational stigma is negative for employee’s work behavior, and reveal the important moderating role of job embeddedness.

## Discussion

Drawing on the JCM and SIP theory, the aim of current study was to investigate the outcomes and boundary conditions of perceived occupational stigma of high-prestige occupations. Using a two-wave field study of full-time preschool teachers in China, we found consistent support for our hypotheses and revealed that perceived occupational stigma is negatively associated with employees’ experienced meaningfulness of their job, while a decrease in experienced meaningfulness is positively associated with employees’ withdrawal behavior. The mediation effect was found to be moderated by employees’ level of job embeddedness. Specifically, preschool teachers who reported higher levels of job embeddedness were more likely to be influenced by perceived occupational stigma. We discuss the theoretical and practical contributions of our study, and future research directions, below.

### Theoretical Implications

The current study offers five main contributions to the literature. First, it presents and tests a framework pertaining to how employees respond to occupational stigma in a high-prestige occupation. As discussed above, researchers have long suggested that almost all occupations are stigmatized to some extent (Hughes, 1951; Kreiner et al., 2006); however, research on stigma pertaining to high-prestige occupations has been lacking. Our research adds empirical evidence to explain the mechanism underlying the influence of occupational stigma on employees in high-prestige occupation (Ashforth and Kreiner, 1999; Kreiner et al., 2006). Interestingly, our findings contradict with a previous study (Baran et al., 2012). Specifically, through an investigation into a low-prestige occupation (animal shelter workers), Baran et al. (2012) found that higher levels of dirty-task involvement are associated with higher levels of job involvement. That is, instead of withdrawing from work, low-prestige occupation workers who conduct dirty work tend to become even more involved with their jobs. However, for high-prestige occupations, our results show that when employees perceived occupational stigma, they tend to withdraw from their work. The reason for this difference may be the different demands for identity management between high (vs low) prestige employees. Low-prestige employees may more eager to construct a positive sense of self, as they suffer both from the dirty tasks and the low occupational prestige (Ashforth and Kreiner, 1999). The dirty work requires them to devote more effort into the job to keep their identification with the work (Baran et al., 2012). However, high-prestige occupation employees do not require this as much as low-prestige occupation employees since they own a high occupation prestige. Actually, this explanation is consistent with our hypotheses about the boundary conditions of job embeddedness. The embeddedness may also indicate the degree of urgency to some extent to keep the identification. It may more urgent for the non-embedded employees (e.g., with lower salaries) to keep their job identification. As a result, the perception of stigma had less impact on them as indicated in our results. Further, Ashforth and Kreiner (1999) suggested that high prestige could serve as a “shield” to buffer the negative influence of occupational stigma on employees. However, few studies have directly examined this assumption. The present study shows that, even behind the “shield” of high prestige, employees suffer negative effects of occupational stigma even when they are embedded in their job.

Second, the current study also extends research by investigating more type of occupational stigma. This is a research gap in this regard that previous studies have called to be filled (Kreiner et al., 2006). Kreiner et al. (2006) developed four types of occupational stigma depending on differences in the breadth (the ratio of dirty work or the centrality of the dirtiness to the occupation’s characteristics) and depth (the intensity of dirtiness and the degree to which workers are in direct contact with it) of dirty work across occupations. Specifically, *pervasive stigma* refers to occupations that are involved with strongly stigmatized tasks or work environments (e.g., miners, prison guards, exotic dancers). *Compartmentalized stigma* refers to occupations in which only parts of tasks are heavily stigmatized (e.g., reporters occasionally have to report on deaths). *Diluted stigma* refers to occupations in which stigma is prevalent but mild (e.g., factory workers who have to work in high temperatures). Finally, *idiosyncratic stigma* represents most occupations in which tasks are neither commonly nor heavily stigmatized. Based on the breadth and depth of dirty work, therefore, preschool teachers may be classified as facing diluted stigma, as the noisy environment (physically tainted) and servile relationship to others (socially tainted) is mild compared to other occupations; or compartmentalized stigma, since child abuse (morally tainted) is occasional but extremely serious. Pervasive stigma has been extensively considered in previous studies; however, research on other types of stigma is limited (Kreiner et al., 2006). Thus, this research contributes to the stigma literature by exploring diluted or/and compartmentalized stigma (Kreiner et al., 2006). In doing so, it more fully explains the influence of stigma associated with various types of occupations.

Third, the study also extends past research by elaborating on the relational mechanisms that mediate occupational stigma and withdrawal behaviors through meaningfulness. Supporting our hypotheses, perceived occupational stigma was found to be negatively associated with meaningfulness, because perceived occupational stigma undermines employees’ ability to perceive the two key job dimensions—skill variety and task significance—that are the antecedents of meaningfulness experience (Hackman and Oldham, 1976; Salancik and Pfeffer, 1977, 1978). Moreover, occupational stigma is likely to convey information to employees that their work is not appreciated by the beneficiaries, and thus it is difficult to perceive the significance of their work, which in turn threatens perceived meaningfulness. We also reveal the mechanisms driving the effect of perceived occupational stigma on withdrawal.

Fourth, following SIP theory (Salancik and Pfeffer, 1978), we outlined an important boundary condition under which occupational stigma is more or less likely to influence meaningfulness and work behavior—job embeddedness. Because prior research on SIP has paid little attention to boundary conditions (Zalesny and Ford, 1990; Yang et al., 2019), this paper contributes to theory by exploring the role of job embeddedness in moderating occupational stigma effects. Our findings suggest that perceived occupational stigma is more likely to negatively influence meaningfulness for employees with higher levels of job embeddedness, who already have sufficient reason to remain in their role (e.g., reasonable salary). However, our results did not show that employees with lower job embeddedness experience greater meaningfulness from their work after perceiving occupational stigma. This may be because some of the non-embedded teachers in our sample came from private kindergartens (non-government-funded) where teachers are primarily recruited from non-preschool education majors. As a result, it may difficult for them to perceive meaningfulness in preschool teaching due to their limited preschool-related education background. Thus, whether or not there is occupational stigma they may tend to experience lower level of meaningfulness. Future research is needed to determine whether employees with different reasons for job embeddedness (or lack thereof) have different responses to occupational stigma.

Fifth, the current findings also have theoretical implications with respect to exploring the negative effect of job embeddedness. The majority of research on job embeddedness has focused on the benefit of embeddedness to individuals and organizations, yet some researchers have also found some adverse effects on employees’ behavior and performance (e.g., Ng and Feldman, 2012; Allen et al., 2016; Juanne et al., 2018). Our research revealed that under situations of occupational stigma, increased embeddedness can injure rather benefit employees; we thus also provide insights into the boundary conditions of embeddedness’ positive effect.

### Practical Implications

This study also presents interesting practical implications. One implication for managerial practice is that perceived occupational stigma can be a negative factor that limits experienced meaningfulness and thus enhances withdrawal behaviors. Therefore, occupational stigma is costly for employees and organizations, as meaningfulness generally has positive effects on job performance (Grant and Campbell, 2007; Grant, 2008), and employees’ withdrawal behavior is harmful to organizations (Hanisch and Hulin, 1991). Thus, both employees and managers should attach importance to this phenomenon and take action to reduce the negative influence of occupational stigma on employees. For employees, the ideological techniques suggested by Ashforth and Kreiner (1999) could be adopted to alleviate the negative influence occupational stigma may have on jobholders; for instance, reiterating positively valued experiences, shifting attention from stigmatized to non-stigmatized features, and so on. On the other hand, for managers, interventions should be implemented to help high-prestige employees to better cope with occupational stigma. For example, in the case of kindergartens managers could hire more full-time or part-time staff to share preschool teachers’ non-teaching tasks in order to help them avoid physically or/and socially tainted job factors as far as possible. In this way, teachers can focus more on children’s education. In addition, as Ashforth et al. (2007) suggested, managers can provide training for employees on how to confront the identity-threatening behaviors occupational stigma may bring about. Moreover, also suggested by Ashforth et al. (2007), managers could provide tour opportunities for the public to dispel public misconceptions about the occupation.

Second, our research identified meaningfulness as a bridge between perceived occupational stigma and withdrawal behavior. Therefore, managers could cultivate employees’ meaningfulness experience via enhancing their perceptions of skill variety and task significance to buffer the impact of occupational stigma on withdrawal. For instance, managers could implement job enrichment programs to make the most of employees’ abilities while increasing the significance of their tasks (e.g., adding curriculum development roles for preschool teachers). This could serve as a signal to employees that their work is significant and valued by the organization and clients, which may help to activate members’ meaningful psychological state (Hackman and Oldham, 1976).

Third, our exploration of the moderating role of job embeddedness demonstrated that the connection between perceived occupational stigma and experienced meaningfulness was stronger for employees with higher job embeddedness. This finding suggests that the negative influence of occupational stigma is contingent on job embeddedness. Being embedded in a stigmatized workplace may be harmful to employees’ meaningfulness, and withdrawal behavior may then be more likely. Thus, consistent with previous research (Allen et al., 2016), we also suggest that organizations could provide more career counseling services or employee assistance programs to employees who are most embedded in the organization, which may help them to cultivate more commitment toward the profession, and eliminate the idea of being “stuck” in the occupation, so as to buffer the negative effects mentioned above.

### Limitations and Recommendations for Future Research

Although the current research has potentially important implications, three primary weaknesses are worth noting. First, the sample comprised preschool teachers, which constrains the generalizability of the findings. Thus, whether these findings apply to other high-prestige occupations, such as physicians, which is another stigmatized high-prestige profession, remains to be tested (Wang et al., 2021). We encourage future studies to replicate our research across more occupations. As one reviewer suggested, it would be interesting to conduct a comparative analysis that includes occupations ranging from those with high to low prestige.

Second, all variables were self-reported by participants, which may raise concerns that some of the observed study relationships were biased by common method variance effects (Podsakoff et al., 2003). However, we gathered information at two different times, which should have reduced the potential influence of common method variance. In addition, preschool teachers usually have some degree of autonomy (Leana et al., 2009). It is thus difficult for other colleagues or leaders to notice all undesirable behaviors (Berry et al., 2012). Therefore, consistent with prior research (Yuan et al., 2021), it may be more appropriate for teachers to self-report their withdrawal behavior. Nevertheless, future research may use objective data (e.g., attendance records) to examine the effect.

Third, drawing on JCM and SIP theory, the present study focused on the mediating effect of meaningfulness; however, only 12% of the variance in meaningfulness was explained even when all variables were included. Further research is thus necessary to identify other mechanisms and outcomes that were omitted from our study. For example, under regulatory focus theory (Higgins, 1997), prevention focus may connect the relationship between occupational stigma and work behaviors. Occupational stigma can be considered a negative work condition, and regulatory focus theory proposes that a prevention focus may be aroused in the face of unfavorable conditions (Higgins, 1997). Thus, occupational stigma may produce a prevention focus in employees, which may lead to less favorable work outcomes.

## Conclusion

By integrating the JCM and SIP theory, this research explored the negative outcomes, relational mechanism, and boundary conditions of occupational stigma. Our research emphasizes the significance of meaningfulness in the occupational stigma–withdrawal behavior relationship, and of job embeddedness as an important contextual boundary condition. As perceived occupational stigma increase, employees with high levels of job embeddedness experience decreased meaningfulness in their work and increased withdrawal behavior, because they have sufficient justification for staying at the stigmatized occupation, and, as a result, the meaningfulness of the job is undermined. Hence, examinations of how employees in high-prestige occupations respond to stigmatized jobs must include inquiries into employees’ level of job embeddedness. Although the current research highlights the connection between occupational stigma and withdrawal behavior, many questions remain to be answered by future research. Nonetheless, we hope the results provided here will spur more research to explore additional mechanisms and outcomes of occupational stigma.

## Data Availability Statement

The raw data supporting the conclusions of this article will be made available by the authors, without undue reservation.

## Ethics Statement

The studies involving human participants were reviewed and approved by the Ethics Committee of Sichuan Normal University. The patients/participants provided their written informed consent to participate in this study.

## Author Contributions

BH contributed to the central idea. BH and LM analyzed most of the data and wrote the initial draft of the manuscript. LM provided the necessary resources for this research. LH collected part of the data, carried out additional analyses, and finalized the manuscript. All authors discussed the results and revised the manuscript.

## Conflict of Interest

The authors declare that the research was conducted in the absence of any commercial or financial relationships that could be construed as a potential conflict of interest.

## Publisher’s Note

All claims expressed in this article are solely those of the authors and do not necessarily represent those of their affiliated organizations, or those of the publisher, the editors and the reviewers. Any product that may be evaluated in this article, or claim that may be made by its manufacturer, is not guaranteed or endorsed by the publisher.

## Appendix

**Appendix Table 1:** Sample measurement items.

Variables	Sample Items
Perceived occupational stigma	Most people who are not preschool teachers have a problem viewing preschool teachers as equals
Job embeddedness	I simply could not leave the organization that I work for
Meaningfulness (Positive meaning)	I have discovered work that has a satisfying purpose
Meaningfulness (Meaning making through Work)	My work helps me better understand myself
Meaningfulness (Greater good motivations)	The work I do serves a greater purpose
Withdrawal (Psychological withdrawal)	Put less effort into job than should have
Withdrawal (Physical withdrawal)	Fallen asleep at work
